# The Role of Overweight and Obesity in In Vitro Fertilization Outcomes of Poor Ovarian Responders

**DOI:** 10.1155/2015/781543

**Published:** 2015-05-27

**Authors:** Fisun Vural, Birol Vural, Yiğit Çakıroğlu

**Affiliations:** ^1^Department of Obstetrics and Gynecology, Haydarpaşa Numune Teaching Hospital, Istanbul, Turkey; ^2^Department of Obstetrics and Gynecology, Division of Reproductive Endocrinology and Infertility, Kocaeli University School of Medicine, Kocaeli, Turkey

## Abstract

*Objective*. Obesity is a worldwide concern with detrimental health effects including decreased fecundity. However, obesity's impact on in vitro fertilization (IVF) is inconclusive and there is little data concerning poor ovarian responders (POR). This study explored the effects of overweight and obesity on IVF outcomes of POR. *Design*. We retrospectively evaluated 188 POR undergoing IVF cycles. *Methods*. Patients were categorized into three groups. Group 1 was normal weight POR (18.5–24.9 kg/m^2^, *n* = 96); Group 2 was overweight POR (25.0–29.9 kg/m^2^, *n* = 52); and Group 3 was obese POR (≥30.0 kg/m^2^, *n* = 40). Main measured outcomes included IVF outcomes. *Results*. The oocyte maturity, total gonadotropin dose-duration, and cycle cancellation rates were similar. Obese women had significantly decreased LH levels. LH < 4 mIU/mL had a sensitivity (62%) and a specificity (86%) for IVF failure (AUC: 0.71). Fertilization rates of obese subjects were significantly lower than normal and overweight subjects (*p* = 0.04). Obese women's clinical pregnancy rates were significantly lower (15%) than normal weight women (33.3%, *p* = 0.01). *Conclusions*. Despite similar counts of recruited mature oocytes, obese POR women had decreased fertilization and clinical pregnancy rates. Obesity rather than overweight significantly decreased IVF outcomes in POR.

## 1. Introduction

The incidence of obesity has risen dramatically worldwide and millions suffer from obesity-related chronic diseases and its adverse metabolic effects. Obesity has been associated with subfertility, and these deleterious effects on reproduction have been recognized since Hippocrates [1]. The impacts of obesity and overweight are manifest in nearly every aspect of female reproductive life from puberty through to pregnancy. Obese women have an increased risk of anovulation, menstrual disturbances, infertility, and polycystic ovarian syndrome (PCOS) [2–5]. Obesity also has an adverse impact on pregnancy outcomes. It is associated with an increased chance of preeclampsia, miscarriage rates, gestational diabetes risk, birth complications, perinatal death, congenital abnormalities of the cardiovascular system, and neural tube defects [6–8]. Previous papers on obesity have reported gonadotropin resistance, diminished oocyte-embryo quality, and implantation failures in IVF patients [2–5]. Despite the bulk of studies, the impact of overweight and obesity on IVF outcomes is inconclusive [9–12].

The goal of IVF is the live birth of an infant, and this issue depends on several factors. Ovarian response is one of the cornerstones of IVF success. In the literature, poor ovarian responses (PORs) are reported in 9−24% of all IVF cycles [13]. After 40 years of experience from the first live IVF baby birth published in 1978, a poor ovarian response is still an important problem [13, 14]. The results in the literature about assisted reproductive technologies (ART) in women with obesity and POR remain inconclusive and conflicting with ongoing scientific discussions [9–12]. Some studies have shown unfavorable ovarian responses to gonadotropins and decreased oocyte retrieval or decreases in embryo quality, implantation rates, clinical pregnancies, and live birth rates in obese women undergoing ART [15–17]. Other studies have not confirmed these results [18–20].

Although the majority of the studies have suggested that obesity is related to POR, there is no research exploring the role of body mass index in poor ovarian responders as determined by the Bologna criteria. In this study, we aimed to explore IVF outcomes in healthy weight, overweight, and obese women with POR.

## 2. Materials and Methods

This was a retrospective study design among women who had undergone IVF/ICSI in Kocaeli University Faculty of Medicine Assisted Reproductive Techniques Unit between May 2011 and December 2013. The clinical and laboratory details of all treatment cycles were prospectively entered into a computer, which were retrospectively retrieved for analysis. We evaluated a cohort of 188 POR women who had undergone an IVF cycle without polycystic ovary syndrome (PCOS), endometriosis, or male factor infertility. The Local Ethics Committee of Kocaeli University approved the study.

Data on patients' characteristic features, body mass index (BMI), and ultrasonographic and laboratory variables were collected from the hospital files. Transvaginal ultrasonography was performed on all patients during the follicular phase to exclude any pelvic pathology and to determine the antral follicle count (AFC, the count of follicles 2–10 mm in size). Day three of hormonal evaluations (serum follicle stimulating hormone (FSH), luteinizing hormone (LH), and estradiol (E2)) and any day of cycle hormonal evaluations (serum antimullerian hormone (AMH), thyroid stimulating hormone (TSH), and free thyroxine (T4) levels) and male semen examination results were obtained from the records.

All patients received a flexible antagonist controlled ovarian hyperstimulation (COH) protocol. Patients were monitored for serum E2, LH, progesterone levels, and serial transvaginal ultrasonographic examinations until the dominant follicle was achieved. Thirty-six hours after ovulation induction with recombinant human chorionic gonadotropin hormone (rhCG, Ovitrelle 250 *μ*g, Serono, Darmstadt, Germany), oocyte retrieval was carried out under sedation-analgesia. Intracytoplasmic sperm injection (ICSI)/IVF was performed for all patients.

The POR definition was done according to the Bologna criteria [14]. This definition includes the following components: (a) women who are over 40 years of age or having other risk factors of POR; (b) a prior stimulation cycle with a POR (≤3 oocytes); and (c) reduced ovarian reserve in tests (i.e., AFC < 5–7 antral follicles or AMH < 0.5–1.1 ng/mL).

Patients were categorized into three groups relative to their BMI. Group 1 was normal weight POR (18.5–24.9 kg/m^2^, *n* = 96), Group 2 was overweight POR (25.0–29.9 kg/m^2^, *n* = 52), and Group 3 was obese POR (≥30.0 kg/m^2^, *n* = 40). Primary outcome measures were MII oocyte counts, fertilization rates, embryo counts, duration of COH, gonadotropin doses, clinical pregnancy rates, and cycle cancelations.

The data were analysed using the Statistical Package for Social Sciences (SPPS) 16 software (SPSS Inc., Chicago, IL, USA) with a 95% confidence interval. The descriptive data were expressed as mean, standard deviation and a percentage. Comparisons of different variables were performed by a one-way analysis of variance (ANOVA) and the *χ* ^2^ test. The relationships between the data were evaluated using Pearson's and Spearman's correlations. The Levene and Welch tests were used to test for homogeneity of variances. Receiver operating curve (ROC) analyses were used for the sensitivity and cut-off values of LH levels.

## 3. Results

This study included a total of 188 POR women aged 22−45 years (34.8 ± 5.1). Group 1 was normal weight POR (*n* = 96), Group 2 was overweight POR (*n* = 52), and Group 3 was obese POR (*n* = 40). The mean age of Group 1 was 35.3 ± 4.8 years while Group 2 was 34.3 ± 4.8 years and Group 3 was 34.3 ± 4.9 years. The comparison of groups concerning ages was similar (*p* > 0.05). The mean duration of infertility in Group 2 and Group 3 was significantly higher than that in Group 1 (*p* = 0.01). However, the duration of infertility in Group 2 and Group 3 was similar (*p* > 0.05). The comparison of age, gravidity, parity, obstetric history including live birth, spontaneous abortion, and ectopic pregnancy was similar between groups (*p* > 0.05).  Table 1 presents the details of this data.

Table 2 shows the comparison of basal hormonal evaluations and antral follicle counts in all groups. FSH, E2, and AMH levels and AFC were similar for all groups (*p* > 0.05). LH levels in Group 3 were significantly lower than those in Group 1 (*p* = 0.00) and Group 2 (*p* = 0.002). The comparison of Group 1 and Group 2 was similar. The ROC analysis showed a cut-off value of 4 mIU/mL for LH levels to predict IVF failure. LH (<4 mIU/mL) yielded a sensitivity of 62% and a specificity of 86% with AUC (0.71) in Groups 2 and 3.

Table 3 shows the comparison of IVF outcomes. MII oocytes, gonadotropin doses, COH duration, and embryo counts were similar between groups. The fertilization rates significantly decreased with BMI (*p* = 0.04). The post hoc analysis showed that Group 3 (64.4%) had significantly decreased fertilization rates compared to Group 1 (82.3%, *p* = 0.008) and Group 2 (79.6%, *p* = 0.01). The fertilization rates were similar between Groups 1 and 2.

The clinical pregnancy rates decreased by BMI (Group 1: 33.3%, Group 2: 25%, and Group 3: 15%; *p* = 0.02). The post hoc analysis showed that Group 3 had significantly reduced clinical pregnancy rates compared to Group 1 (*p* = 0.01), but the decreases between Group 2 and Group 3 did not reach statistical significance (*p* > 0.05).

## 4. Discussion

A poor ovarian response may be the first sign of ovarian aging since COH is the dynamic test showing the ovarian follicular pool [14, 21, 22]. Due to a lack of uniformity in the definition, the exact incidence of POR is difficult to estimate. Although the term POR has been used over 30 years, a standardized definition could not be achieved through the 2011 ESHRE consensus [14]. There is conflicting data to conclude the role of obesity in IVF outcomes [9–12]. Although the majority of studies have suggested that obesity is related to POR, there is no study that has examined the impact of BMI on IVF outcomes with POR determined with the Bologna criteria. This paper investigated the impact of BMI on IVF outcomes in POR. This study has shown that obesity rather than overweight in POR adversely affects IVF outcomes.

In this paper, obese women had reduced LH levels, but FSH, AMH, and E2 levels and total antral follicle counts were similar (*p* > 0.05). The decreased LH levels in obese women confirmed previous results. The reduced LH levels may be partially explained by an increased aromatization of androgens to oestrogens that suppress LH secretion from the hypothalamic-hypophyseal system [23, 24]. Obesity excluding PCOS is a relative hypogonadotropic hypogonadism state with an unknown exact physiology. Obese people have hyperleptinemia that causes central leptin resistance and hypogonadism; such a mechanism could explain the altered pulsatile LH amplitude in obese women [23, 24]. However, the use of aromatase inhibitors in POR patients is based on little evidence [25].

Ovarian follicular development is regulated by intraovarian regulators, and the two-cell theory suggests that both FSH and LH are necessary for normal follicular growth [26]. The secretion of these regulatory proteins occurs under gonadotropin stimulation. It is speculated that when day three LH levels are less than 3 mIU/mL, there may be reduced activity of these ovarian regulators, thus fewer counts of preovulatory follicles [27]. Similarly, in this study, obese POR with LH levels less than 4 mIU/mL had an 86% specificity of predicting IVF failure. Future prospective randomized controlled trials may demonstrate whether alternative therapies for stimulation, such as aromatase inhibitors or LH treatment, can increase the pregnancy rates.

The complex events during follicular maturation predict the capacity of oocyte fertilization and subsequent embryonic development. Thus, fertilization rates are one of the markers of oocyte quality [1]. In this study, despite similar mature oocyte counts in groups, fertilization rates were significantly decreased in the obese group. This suggests to us that the oocyte quality is decreased in obese women. Despite the bulk of previous studies, some researchers have found reduced fertilization rates in obese women [2–5], but others have not [18–20].

Previous studies concerning obesity and the endometrium have reported contradictory results [28–30]. The donor oocyte study has been proposed as a model to investigate endometrial receptivity [28]. Recently, Jungheim et al. published the first systematic review and meta-analysis addressing this topic; they stated that obesity does not affect IVF outcomes in women using donor oocytes [29]. Another recent study by Bellver et al. suggested decreased uterine receptivity of obese and ovum donated women in their study of 9,587 egg donations [30]. Thus, a scientific discussion about obesity and endometrial receptivity is going on. In our study, despite similar ratios of mature oocyte counts obtained in all weight groups, decreased fertilization and clinical pregnancy rates are suggested as factors related to decreased oocyte quality related to reduced fertility rates. However, future studies with endometrial receptivity in autologous cycles are needed.

A previous report showed the impact of weight reduction on reproductive function [23]. The results of our study suggested that POR women with obesity should receive counsel for weight reduction programs before an IVF trial. However, the effect of weight reduction on IVF outcomes of POR women needs further prospectively designed studies.

This paper has some limitations due to the retrospective design because smoking habits and male factor obesity are unknown. Strengths of this study include the use of the Bologna criteria for POR and the fact that we excluded male factor infertility, endometriosis, and PCOS cases that could affect IVF outcomes. Thus, the results are comparable for future standardized research. To the best of our knowledge, this is the first study comparing IVF outcomes of obese, overweight, and healthy weight POR by using ESHRE criteria.

In conclusion, despite obtaining similar mature oocyte counts, fertilization and clinical pregnancy rates significantly decreased in the obese group. This suggests that obesity, rather than overweight, adversely affects IVF outcomes in women with poor ovarian response. Future prospective randomized controlled trials may enlighten whether weight reduction affects the outcome or whether alternative therapies for stimulation such as aromatase inhibitors or LH treatment can increase pregnancy rates.

## Figures and Tables

**Table 1:** The characteristic findings of women (mean ± standard deviation).

	Group 1	Group 2	Group 3	*p* value
Age	35.3 ± 4.8	34.3 ± 4.8	34.3 ± 4.9	0.468
Gravidity	0.3 ± 0.7	0.5 ± 0.9	0.4 ± 0.6	0.267
Parity	0.03 ± 0.18	0.02 ± 0.16	0.12 ± 0.4	0.228
Abortion	0.18 ± 0.54	0.4 ± 0.64	0.18 ± 0.39	0.115
Live birth	0.01 ± 0.13	0.01 ± 0.0	0.06 ± 0.24	0.224
Ectopic pregnancy	0.11 ± 0.45	0.16 ± 0.5	0.06 ± 0.21	0.291
Infertility duration (years)	5.6 ± 4.1	7.6 ± 5.0	9.3 ± 5.2	**0.01**

**Table 2:** The basal hormonal and ultrasonographic characteristics (mean ± standard deviation).

	Group 1	Group 2	Group 3	*p* value
FSH (mIU/mL)	9.4 ± 5.7	8.0 ± 3.1	7.5 ± 3.9	0.146
LH (mIU/mL)	5.7 ± 2.6	5.5 ± 3.2	3.5 ± 1.6	**0.015**
E2 (pg/mL)	58.4 ± 53.0	45.3 ± 26.3	50.9 ± 30	0.352
AMH (ng/mL)	0.7 ± 1.4	0.8 ± 0.8	1.1 ± 1.2	0.775
Antral follicle count	7.0 ± 4.0	9.1 ± 6.2	8.2 ± 4.0	0.213

**Table 3:** The comparison of groups with respect to IVF outcomes.

	Group 1	Group 2	Group 3	*p* value
M_2_ oocyte counts	1.7 ± 0.9	1.7 ± 0.8	1.9 ± 1.0	0.461
Fertilization rates (%)	82.3 ± 32.2	79.6 ± 33.4	64.4 ± 34.4^a,b^	**0.04**
Embryo counts	1.5 ± 1.0	1.3 ± 0.8	1.5 ± 1.0	0.685
Stimulation duration (days)	8.5 ± 2.5	8.6 ± 2.0	8.6 ± 3.1	0.977
Gonadotropin dose (units)	2993 ± 1212	2772 ± 844	2752.8 ± 1003	0.546
Clinical pregnancy rates (%)	33.3	25	15^c^	**0.02**
Cycle cancellation rates (%)	16.6	17.3	20	0.732

Post hoc tests: ^a^Group 2 versus Group 3, *p* = 0.01; ^b^Group 3 versus Group 1, *p* = 0.008; ^c^Group 3 versus Group 1, *p* = 0.01.
